# Detection of *EGFR* mutations in circulating free DNA by PNA-mediated PCR clamping

**DOI:** 10.1186/1756-9966-32-50

**Published:** 2013-08-09

**Authors:** Hye-Ryoun Kim, Sung Yong Lee, Dae-Sung Hyun, Min Ki Lee, Hyun-Kyung Lee, Chang-Min Choi, Sei-Hoon Yang, Young-Chul Kim, Yong Chul Lee, Sun Young Kim, Seung Hun Jang, Jae Cheol Lee, Kye Young Lee

**Affiliations:** 1 Korea Cancer Center Hospital, Seoul, Korea; 2 Korea University Guro Hospital, Seoul, South Korea; 3 Daegu Catholic University Medical Center, Daegu, Korea; 4 Pusan National University Hospital, Busan, Korea; 5 Inje University Busan Paik Hospital, Busan, South Korea; 6 Department of Oncology, Asan Medical Center, College of Medicine, University of Ulsan, 388-1 Pungnap-2 Dong, Songpa-Gu, Seoul 138-736, Korea; 7 Wonkwang University Hospital, Iksan, Korea; 8 Chonnam National University Hwasun Hospital, Hwasun-Gun, Korea; 9 Chonbuk National University Hospital, Chonju, Korea; 10 Chungnam National University Hospital, Daejeon, Korea; 11 Hallym University Sacred Heart Hospital, Anyang, Korea; 12 Department of Internal Medicine, Konkuk University Medical Center, 4-12 Hwayang-dong, Gwangjin-gu, Seoul 143-729, Korea

**Keywords:** Plasma, *EGFR* mutation, PNA-mediated PCR clamping method, Non-small cell lung cancer

## Abstract

**Background:**

Epidermal growth factor receptor (*EGFR*)-activating mutations are major determinants in predicting the tumor response to EGFR tyrosine kinase inhibitors in non-small cell lung cancer (NSCLC). Noninvasive test for the detection of *EGFR* mutations is required, especially in NSCLC patients from whom tissue is not available. In this study, we assessed the feasibility of detection of *EGFR* mutations in free DNA circulating in plasma.

**Methods:**

Plasma samples of 60 patients with partial response to gefitinib were analyzed to detect *EGFR*-activating mutations in exons 19 and 21. Forty (66.7%) of patients had tumor *EGFR* mutation results. *EGFR* mutations in plasma were detected using the peptide nucleic acid (PNA)-mediated polymerase chain reaction (PCR) clamping method. All clinical data and plasma samples were obtained from 11 centers of the Korean Molecular Lung Cancer Group (KMLCG).

**Results:**

Of the 60 patients, 39 were female and the median age was 62.5 years. Forty-three patients never smoked, 53 had adenocarcinomas, and seven had other histologic types. *EGFR*-activating mutation was detected in plasma of 10 cases (exon 19 deletion in seven and exon 21 L858R point mutation in three). It could not be found in plasma after treatment for 2 months. When only patients with confirmed *EGFR* mutation in tumor were analyzed, 17% (6 of 35) of them showed positive plasma *EGFR* mutation and the mutation type was completely matched with that in tumor. There was no statistically significant difference in clinical parameters between patients with *EGFR* mutations in plasma and those without *EGFR* mutations.

**Conclusions:**

The detection rate of *EGFR* mutations from plasma was not so high despite highly sensitive *EGFR* mutation test suggesting that more advances in detection methods and further exploration of characteristics of circulating free DNA are required.

## Background

Epidermal growth factor receptor (*EGFR*) mutations, such as deletions in exon 19 and point mutations in exon 21, are considered the most reliable predictive factors of outcome after treatment of non-small cell lung cancer (NSCLC) with EGFR tyrosine kinase inhibitors (EGFR-TKIs). Gefitinib was approved as a first-line therapy for NSCLC based on the results of a phase III landmark study, the Iressa Pan-Asia Study (IPASS), which showed that gefitinib conferred a survival benefit in *EGFR* mutation-positive patients over conventional chemotherapy [1]. The trial clearly showed that the selection of EGFR-TKIs should be based on molecular markers, not on clinical characteristics. Since then, given that many patients cannot receive second-line therapy after first-line failure because of their generally deteriorating condition, *EGFR* mutation testing is requested more frequently at the time of diagnosis for patients with adenocarcinoma. Indeed, a European workshop on *EGFR* mutation testing in NSCLC recommended testing at diagnosis, or at relapse, whenever possible, although no gold standard testing method was chosen [2].

Despite their importance in clinical practice, there is often too little tissue available to examine *EGFR* status as most are obtained by small needle biopsy or extracted from body fluids rather than via a more aggressive surgical approach. Many investigators have tried to solve this problem, leading to the development of more sensitive techniques to detect *EGFR* mutations, such as the scorpion-amplified refractory mutation system (SARMS) and the peptide nucleic acid (PNA)-mediated polymerase chain reaction (PCR) clamping method [3-18]. In addition, it is suggested that the plasma of cancer patients contains circulating free DNA (cfDNA) originating from necrotic tumor cells sloughed from the tumor mass or from circulating tumor cells [19-21]. Attempts to detect *EGFR* mutations in cfDNA using these sensitive techniques are currently in progress. If proven feasible and reliable, the cfDNA test may have broad clinical applications because it is non-invasive, convenient and can be performed repeatedly. In addition, the test could help diagnose lung cancer in cases when an adequate tissue sample is difficult to obtain. Over the past several years, many reports have shown promising results and have supported the feasibility of the test [22-33]. However, the optimal methodology for mutation detection from cfDNA and the possibility for the replacement of tumor tissue to blood sample still need to be confirmed.

In the present study, we examined the status of *EGFR* mutations in cfDNA isolated from plasma samples by a PNA-mediated PCR clamping method (PNA test) to determine the utility of plasma as a surrogate tissue for *EGFR* mutation analysis.

## Methods

### Patients

The prospective multicenter study was conducted to analyze *EGFR* mutations in plasma samples. Sixty patients with advanced NSCLC were recruited from 11 hospitals of the Korean Molecular Lung Cancer Group (KMLCG) between May 2010 and March 2011. All participants had histological or cytological confirmation of advanced NSCLC and showed a partial response to gefitinib as a second-line therapy without regard to the *EGFR* mutation status. Written informed consents for the use of their blood were obtained from all patients. The study protocol was approved by the Ethical Review Committee of 11 institutions (Korea Cancer Center Hospital, Korea University Guro Hospital, Daegu Catholic University Medical Center, Pusan National University Hospital, Inje University Busan Paik Hospital, Asan Medical Center, Wonkwang University Hospital, Chonnam National University Hwasun Hospital, Chonbuk National University Hospital, Chungnam National University Hospital, Hallym University Medical Center, Konkuk University Medical Center).

### Plasma sample collection and DNA extraction

Whole blood specimens from patients were collected in ethylenediaminetetraacetic acid tubes before and 2 months after the initiation of gefitinib administration and centrifuged at 3000 rpm for 5 minutes. The supernatants were collected and centrifuged at 3000 rpm for 5 minutes. The final supernatants were transferred to Eppendorf tubes and stored at −70°C until DNA extraction. DNA was extracted from 1–2 ml of supernatant with a DNeasy blood kit (Qiagen) according to the manufacturer’s instructions. The final elution volume for DNA extraction was 60 μl and the amount of plasma DNA used for mutation testing was 30 ng.

### PNA-mediated real-time PCR clamping method to detect deletions in EGFR exon 19 and L858R point mutations in EGFR exon 21

Plasma DNA was analyzed using the PNAClamp^TM^ EGFR Mutation Detection kit (PANAGENE, Inc., Daejeon, Korea) as described in a previous retrospective study [34]. All reactions were conducted in a 20-μl volume using template DNA, primers and PNA probe set, and SYBR Green PCR master mix. All reagents were included in the kit. Real-time PCR reactions were performed using a CFX 96 instrument (Bio-Rad, USA). PCR cycling commenced with a 5 min hold at 94°C followed by 40 cycles at 94°C for 30 s, 70°C for 20 s, 63°C for 30 s, and 72°C for 30 s. Two *EGFR* mutation types were detected using PNA-mediated real-time PCR. The efficiency of PCR clamping was determined by measuring the cycle threshold (Ct) value. Ct values for the control and mutation assays were obtained by observing the SYBR Green amplification plots. The delta Ct (∆Ct) value was calculated (control Ct − sample Ct), ensuring that the sample and control Ct values were from the test and wild-type control samples. The cut-off ∆Ct was defined as 2 for both the G746_A750 deletion and the L858R point mutation.

### Tumor mutation data

At time of blood collection, we reviewed the *EGFR* mutation status in patient matched tumor tissue. By the direct sequencing used in routine practice at each institution to established *EGFR* mutation status in tumor tissue, forty tumor specimens were analyzed for *EGFR* mutations before gefitinib.

### Statistical analyses

The relationship between *EGFR* mutations and demographic and clinical features, including age, gender, histological type, performance status (PS), smoking status, TNM stage and response to gefitinib, was analyzed using Pearson’s chi-square test or Fisher’s exact test. Two-sided P values <0.05 were considered statistically significant. All analyses were performed using SPSS version 17.0 (SPSS Inc., Chicago, IL, USA).

## Results

### Patient characteristics

The clinical characteristics of the 60 patients are shown in Table 1. The median age was 62.5 years (range: 38–84 years). Thirty-nine (65.0%) of the patients were female and 21 (35.0%) were male. Forty-three patients (71.7%) were non-smokers. Fifty patients (83.3%) had good PS. The most common histological subtype was adenocarcinoma (53 patients, 88.3%) and the majority of patients (88.3%) had stage IV disease. As aforementioned, all the patients received second-line gefitinib treatment and showed partial response.

**Table 1:** Clinical characteristics of 60 patients

	**Total (n = 60)**
Age	
Median, years	62.5
Range	38-84
Gender	
Female	39 (65.0%)
Male	21 (35.0%)
Smoking history	
Nonsmoker	43 (71.7%)
Ex-smoker	11 (18.3%)
Current smoker	6 (10.0%)
WHO Performance status	
Normal activity	23 (38.3%)
Restricted activity	27 (45.0%)
In bed < 50% of the time	9 (15.0%)
In bed > 50% of the time	1 (1.7%)
Tumor histology	
ADC	53 (88.3%)
SQC	3 (5.0%)
LCC	1 (1.7%)
NSCLC NOS	2 (3.3%)
Others	1 (1.7%)
Stage	
IIIA	3 (5.0%)
IIIB	4 (6.7%)
IV	53 (88.3%)

Abbreviations: *ADC* adenocarcinoma, *SQC* squamous cell carcinoma, *LCC* large cell carcinoma, *NSCLC NOS* non-small cell lung cancer not otherwise specified.

### Detection of EGFR mutations in plasma

*EGFR* mutations were identified in 10/60 (16.7%) plasma samples by PNA testing. Of these, seven (70.0%) were in-frame deletions within exon 19 and three (30.0%) were arginine-to-leucine substitutions at amino acid 858 in exon 21 (L858R) (Table 2). After 2 months of treatment, a repetition of the test in *EGFR* mutation-positive patients showed that none had *EGFR* mutations.

**Table 2:** EGFR mutational status in plasma DNA samples

	**Positive**	**Negative**
	**EGFR mutation**	**EGFR mutation**
	**(n = 10)**	**(n = 50)**
Exon 19 deletion	7 (70.0%)	-
Exon 21 point mutation	3 (30.0%)	-

### Comparison of matched tumor sequencing and plasma EGFR mutations

To evaluate the accuracy of the results of the PNA test, we compared plasma *EGFR* mutations with tumor sequencing in 40 paired donor-matched plasma and tumor tissue specimens. *EGFR* mutations were detected in the plasma samples of six (15.0%) patients, including four deletions in exon 19 and two point mutations in exon 21. In the donor-matched tumor tissues, 35 mutations were detected (87.5%) by using direct sequencing, including 18 in exon 19 and 17 in exon 21. Of the patients with plasma *EGFR* mutations, mutations of identical exon site were detected in the matched tumor tissues (Table 3).

**Table 3:** EGFR mutational status in the paired specimens of plasma and tumor tissue

**N = 40**		**Plasma EGFR mutation**	
		**Positive**	**Negative**
**Tissue EGFR mutation**	**positive**	6	29
	**negative**	0	5

### Correlation between EGFR mutation status assessed by PNA-mediated real-time PCR clamping and clinical features

*EGFR* mutations in plasma were detected more frequently in females (17.9% vs. 14.3% in male), non-smokers (18.6% vs. 11.8% in current/former smokers) and patients with stage IIIB disease (25.0% vs. 17.0% in stage IV). In addition, the overall mutation detection rate at the institute at which the central laboratory was located, and where sample processing did not require shipment, was relatively higher than that at the other institutes (23.8% vs. 12.8%); however, there were no statistically significant differences between the number of patients with *EGFR* mutations in plasma and those without (Table 4).

**Table 4:** Different characteristics according plasma EGFR mutational status

	**Patients with plasma EGFR mutation**	**Patients without plasma EGFR mutation**	***P *value**
	**(n = 10)**	**(n = 50)**	
Age			
Median, years	60.5	63.0	0.76
Range	51-76	38-84	
Gender			
Female	7 (70.0%)	32 (64.0%)	1.00
Male	3 (30.0%)	18 (36.0%)	
Smoking history			
Nonsmoker	8 (80.0%)	35 (70.0%)	0.67
Ex-smoker	1 (10.0%)	10 (20.0%)	
Current smoker	1 (10.0%)	5 (10.0%)	
WHO Performance status			
Normal activity	4 (40.0%)	19 (38.0%)	0.94
Restricted activity	4 (40.0%)	23 (46.0%)	
In bed < 50% of the time	2 (20.0%)	7 (14.0%)	
In bed > 50% of the time	-	1 (2.0%)	
Tumor histology			
ADC	9 (90.0%)	44 (88.0%)	0.83
SQC	-	3 (6.0%)	
LCC	-	1 (2.0%)	
NSCLC NOS	1 (10.0%)	1 (2.0%)	
Others	-	1 (2.0%)	
Stage			
IIIA	-	3 (6.0%)	0.64
IIIB	1 (10.0%)	3 (6.0%)	
IV	9 (90.0%)	44 (88.0%)	
Central labotory			
on-site	5 (50.0%)	16 (32.0%)	0.30
off-site	5 (50.0%)	34 (68.0%)	

Abbreviations: *ADC* adenocarcinoma, *SQC* squamous cell carcinoma *LCC* large cell carcinoma, *NSCLC NOS* non-small cell lung cancer not otherwise specified.

## Discussion

Direct sequencing of amplified DNA products using Sanger’s method is the most popular test for detecting *EGFR* mutations. However, this method is limited by low sensitivity (meaning that the mutant DNA must represent greater than 25% of the total DNA), and requires multiple steps to be performed over several days [15]. Furthermore, in patients with advanced NSCLC, tumor tissue is not always available for *EGFR* mutation testing either because only small amounts of tissue are collected or because the tissues collected have very low, or non-existent, tumor content . For these reasons, new techniques are needed for more sensitive and rapid detection. Several new techniques, including SARMS, Taqman PCR, and denaturing high-performance liquid chromatography (dHPLC) have been introduced, although none have been adopted as a standard method for detecting *EGFR* mutations [4,5,9-11,13,14,16,22-24,26-28],[30-33].

Peptide nucleic acid (PNA) is an artificial polymer with the properties of both nucleic acids and proteins. PNA can bind tightly to complementary sequences in DNA because of a lack of electrostatic repulsion. Therefore, when a PNA oligomer, designed to detect an *EGFR* mutation and to bind to the antisense strand of the wild-type *EGFR* gene, is used for real-time PCR, amplification is rapid and sensitive and displays similar sensitivity to SARMS. Several studies using this novel method have been published [8,17,34,35], however, to our knowledge, there are no reports showing detection of *EGFR* mutations in cfDNA extracted from the plasma of NSCLC patients using PNA-mediated real time PCR clamping.

In the present study, the detection rate of *EGFR* mutations in cfDNA was 16.1%. This is somewhat lower than that reported previously, which ranges from 20% to 73% (Table 5) [16,24,26-28,32]. Mutation detection rates can differ between subjects and between methods. Around 50–60% of Asian patients and 20–30% of Western patients with adenocarcinomas are expected to carry activating *EGFR* mutations, while a negligible proportion of patients with other lung cancer histology are expected to carry such mutations. Therefore, the *EGFR* mutation detection rate can be estimated from the clinical and demographic parameters, including race and histology, of the study subjects. If we assume that 50% of Asian adenocarcinoma patients carry *EGFR* mutations, the expected detection rate in an Asian study population comprising 80% adenocarcinoma patients should be 40%. In this context, the results of several previous studies suggesting that the *EGFR* mutation test in cfDNA might be equivalent to that in tissue exceed the expected rate of *EGFR* positivity. Hence, it is difficult to accept these although the tests used in those studies are highly sensitive and always performed with the utmost precision. In addition, other reports published detection rates around 20% [26,27], which is similar to our report, and still *EGFR* mutation testing in cfDNA has not been introduced in clinical practice in spite of such promising results over several years. Therefore, more data are required to evaluate the suitability of the cfDNA test and assess whether it can replace the traditional tumor tissue test.

**Table 5:** Previous reports on EGFR mutation test from circulating free DNA

**Year**	**Authors**	**Subjects**	**DNA concentration**	**Mutation test**	**Detection rate**
2006	Kimura H, et al. [16]	Asian	70 ng/mL (range, 0–1720 ng/mL)	SARMS	48.1% (13/27)
		Female : 37%			
		Nonsmoker : N/A			
		ADC : 85%			
		ORR : 33%			
2008	Maheswaran S, et al. [24]	Western	N/A	SARMS	39% (7/18)
		EGFR mutant patients			
2009	He C, et al. [29]	Asian	N/A	Mutant-enriched PCR	49.3% (66/134)
		Female : 37%			
		Nonsmoker : 53%			
		ADC : 75%			
2009	Bai H, et al. [28]	Asian	N/A	dHPLC	34.3% (79/230)
		Female : 46%			
		Nonsmoker : 55%			
		ADC : 74%			
		ORR : 36% (37/102)			
2009	Mack PC, et al. [26]	Western/Asian : 96/4%	2.3 ng/μL (range, 1–9 ng/μL )	SARMS	20% (10/49)
		Female : 56%			
		Nonsmoker : 53%			
		ADC : 67%			
2009	Kuang Y, et al. [25]	Western	52.3 ng/μL (range, 10–163 ng/μL )	SARMS and WAVE/Surveyor	54% (29/54)
		Female : 81.5%			
				Whole genome amplification	
		Nonsmoker : N/A			
		ADC : N/A			
		ORR : 56%			
2010	Brevet M, et al. [31]	Western	N/A	Mass spectrometry genotyping assay (Sequenom) and mutant-enriched PCR Whole genome amplification	23.2% (10/31)
		Female : 52%			
		Nonsmoker : 45%			
		ADC : 97%			
2010	Jian G, et al. [27]	Asian	N/A	Taqman PCR	23.2% (13/56)
		Female : 46%			
		Nonsmoker : 58%			
		ADC : 78%			
		ORR : 30%			
2011	Jiang B, et al. [30]	Asian	Minimum 4 ng/μL (range, 11–66 ng/μL )	Mutant-enriched PCR	31% (18/58)
		Female : 31%			
		Nonsmoker : 38%			
		ADC : 72%			
2011	Taniguchi K, et al. [32]	Asian	N/A	BEAMing	72.7% (32/44)
		EGFR mutant patients			
This study	Kim HR, et al.	Asian	8.6 ng/μL	PNA-based PCR clamping	16.7% (10/60)
		Female : 65%			
		Nonsmoker : 2%			
		ADC : 88%			
		ORR : 100%			

Abbreviations: *ADC* adenocarcinoma, *SARMS* scorpion-amplified refractory mutation system, *PCR* polymerase chain reaction, *dHPLC* denaturing high-performance liquid chromatography, *BEAMing* beads, emulsion, amplification, and magnetics, *PNA* peptide nucleic acid.

The T790M mutation was not detected in any of the samples that were positive for activating *EGFR* mutations, although one report showed that low levels of T790M were detected in pretreatment tumor samples from 10/26 patients (38%) [24]. The detection rate of T790M seems to be closely associated with the sensitivity of the *EGFR* mutation test. A study using the BEAMing (beads, emulsion, amplification, and magnetics) method showed that the proportion of T790M within activating mutations ranged from 13.3–94.0%, and calculated that the T790M peak within the mutant allele fraction would range from 0.1–1% in cfDNA [32]. Therefore, even with a higher sensitivity permitting detection of 1% mutant DNA, as is reached with SARMS and PNA-based PCR clamping, detection of the T790M mutation in cfDNA remains difficult. This suggests that circulating tumor cells (CTC) would be a better alternative source material in which to detect the T790M mutation, and for predicting progression-free survival.

None of the *EGFR* mutations initially detected in cfDNA before treatment were detected 2 months after EGFR-TKI therapy and partial response. Since the initial tumor size and stage did not correlate with the detection rate, this result suggests that the amount of actively proliferating tumor cells, rather than the tumor burden, could affect the amount of circulating tumor DNA. Accordingly, in a previous CTC study, a 50% decline in CTCs within 1 week was noted in one patient, with the nadir reached 3 months after treatment, while the number of CTCs increased at the time of clinical progression and declined again when the tumor responded to subsequent chemotherapy [24]. It was also evident that, although CTC detection was not associated with initial tumor burden, there was a close concordance between tumor response and the number of CTCs during treatment.

Finally, our results suggest that better processing of plasma samples and on-site testing without necessity of sample delivery can improve detection rate.

In summary, our results show that, although detection of *EGFR* mutations in cfDNA is possible in some patients, more data are required to evaluate clinical applicability. Technical advances in sensitivity, stability and standardization are also needed, as well as adequate sample processing.

## Competing interests

The authors had no competing interest to declare.

## Authors’ contributions

YCK, SHJ, KYL and JCL contributed to study conception and design. SYL, DSH, MKL, HKL, CMC, SHY, YCK and SYK were involved in acquisition and analysis of data, HRK and JCL wrote the manuscript. KYL confirmed the final draft. All authors read and approved the final manuscript.
